# Improving sampling of crystallographic disorder in ensemble refinement

**DOI:** 10.1107/S2059798321010044

**Published:** 2021-10-20

**Authors:** Nicoleta Ploscariu, Tom Burnley, Piet Gros, Nicholas M. Pearce

**Affiliations:** a Structural Biochemistry, Bijvoet Centre for Biomolecular Research, Utrecht University, Padualaan 8, 3584 CH Utrecht, The Netherlands; b Scientific Computing Department, Science and Technology Facilities Council, Research Complex at Harwell, Didcot OX11 0FA, United Kingdom; c Chemistry and Pharmaceutical Sciences, Free University of Amsterdam, Amsterdam, The Netherlands

**Keywords:** ensemble refinement, structure refinement, molecular dynamics, disorder modelling

## Abstract

Ensemble refinement, the application of molecular dynamics to crystallographic refinement, explicitly models the disorder inherent in macromolecular structures. These ensemble models have been shown to produce more accurate structures than traditional single-model structures. However, suboptimal sampling of the molecular-dynamics simulation and modelling of crystallo­graphic disorder has limited the utility of the method, and can lead to unphysical and strained models. Here, two improvements to the ensemble refinement method implemented within *Phenix* are presented: DEN restraints, which guide the local sampling of conformations and allow a more robust exploration of local conformational landscapes, and ECHT disorder models, which allow the selection of more physically meaningful and effective disorder models for parameterizing the continuous disorder components within a crystal. These improvements lead to more consistent and physically interpretable simulations of macromolecules in crystals, and allow structural heterogeneity and disorder to be systematically explored on different scales. The new approach is demonstrated on several case studies and the SARS-CoV-2 main protease, and demonstrates how the choice of disorder model affects the type of disorder that is sampled by the restrained molecular-dynamics simulation.

## Abstract 2

Improvements to the ensemble refinement method are described and demonstrated. These improvements lead to more physically meaningful and interpretable macromolecular ensembles.

## 1. Introduction   

Disorder in crystallographic data arises from both distinct *discrete* atomic configurations – alternate conformations – and *continuous* variations around the average positions of atoms in each of these conformations. Whereas discrete alternate conformations correspond to different energy minima of a system, the extent of continuous local variations reflect the shape of the local energy landscape (the sharpness or shallowness of the energy minimum). For an atomic model to accurately reflect experimental crystallographic data, both sources of disorder must be represented: the model must contain atomic configurations that sample all of the distinct energy wells, but must also fully represent the shape of each energy well. In typical atomic models, the discrete disorder is modelled with alternate conformations (Keedy *et al.*, 2015 ▸; van Zundert *et al.*, 2018 ▸), while the continuous disorder is modelled with *B* factors. The *B*-factor models that are used in macromolecular refinement typically only account for harmonic oscillations around an average position, and therefore if the oscillation is anharmonic, *B* factors will only give an approximate solution. Any anharmonic oscillations can also be modelled through the use of alternate conformations, but this is rarely performed for standard refinements because the addition of multiple alternate conformations rapidly increases the number of model parameters, leading to overparameterization and overfitting.

Atomic disorder in crystals arises from motions on multiple different length scales: atoms may move independently relative to their surroundings, but collective motions can also exist for individual residues, loops and secondary-structure elements, or even entire domains or molecules. This leads to a layered system of motions on top of motions. Each of these motions will lead to a distinct disorder component. Therefore, to properly characterize and analyze crystallographic disorder, a multi-component disorder model is required that contains elements for each different source of disorder.

The ECHT disorder model (Pearce & Gros, 2021 ▸) is a hierarchical disorder model composed of multiple levels, where each level contains different TLS (translation–libration–screw; Winn *et al.*, 2001 ▸) partitions for possible collective motions of arbitrary groups of atoms (for example molecular motions or residue motions). By using an elastic net approach (Zou & Hastie, 2005 ▸), a parsimonious disorder model can be obtained that assigns disorder to the appropriate length scale in the crystal. We can therefore partition the atomic disorder in our model into collective molecular disorder, collective secondary-structure motions, collective residue motions and finally atomic motions that are not described by the rigid-body approximation. In optimization of the ECHT model, disorder components are implicitly assumed to be independent, which although not strictly valid is a useful approximation. Under the approximation that disorder components are independent, they are additive, and thus an arbitrary collection of motions can be represented by summing together the relevant components.

Ensemble refinement (ER) is a method for structure determination that samples alternate conformations through molecular-dynamics (MD) simulations that are restrained by crystallographic data (Burnley *et al.*, 2012 ▸). The target of the simulation is to use time-averaged crystallographic restraints (Gros *et al.*, 1990 ▸), which guide the conformational sampling to fit the experimental data, while simultaneously allowing the discovery of minor conformations of macromolecules by allowing the simulations to escape from local minima. An important effect of the time-averaged restraints is that if a simulation has recently fully sampled a particular conformation (that according to the data is partially occupied), it is driven away from this location and encouraged to sample other states. Therefore, the utilization of MD simulations allows the atomic model to explore the disorder in the structural data in a proportionate but unbiased fashion, and has been shown to be successful in creating more accurate structural models (as measured by *R*_free_) for high-resolution crystallographic data sets (Burnley *et al.*, 2012 ▸). These structures have proved to be useful in exploring protein dynamics within crystals, with applications in studies of conformational dynamics (Otten *et al.*, 2018 ▸; Campbell *et al.*, 2016 ▸), in studies of TCR–peptide–MHC complexes (Fodor *et al.*, 2018 ▸; Buckle & Borg, 2018 ▸; Loll *et al.*, 2020 ▸) and recently in probing the temperature dependence of structures of the SARS-CoV-2 main protease (Ebrahim *et al.*, 2021 ▸).

As discussed above, crystallographic disorder is the sum of many disorder components from many distinct motions. All of these different disorder components must be represented in the atomic model to accurately reflect the experimental data, but sampling all disorder components in ER requires long simulations. To reduce the amount of disorder that the molecular-dynamics simulation must sample, ER uses TLS-derived models to account for large-scale (typically molecule-scale) disorder components. The MD simulation must then only account for local disorder; this simultaneously reduces the required length of the MD simulation while also improving the quality of the model.

However, the current quality and efficiency of these simulations is limited by multiple factors: the quality of the molecular-dynamics force field, the length scale of the simulation and the quality of the disorder model that is used to complement the MD simulation. These limitations can be broadly summarized in two categories: search problems, where the electron density is not appropriately sampled during the simulation, and physicality problems, where the output model is not a thermodynamically realistic ensemble of conformations. In this work, we present two improvements to the ER method which begin to address these concerns: the incorporation of deformable elastic network (DEN) restraints (Schröder *et al.*, 2010 ▸), which stabilize and improve the sampling of the MD simulation, thus overcoming much of the current search problem, which is exhibited chiefly in the most disordered parts of the models, and the usage of improved disorder models extracted from the ECHT disorder analysis method (Pearce & Gros, 2021 ▸), which overcome imbalances in the distribution of heterogeneity over models, such as the ‘freezing out’ of the centres of macromolecules.

## 2. Incorporation of DEN restraints and ECHT disorder models within ensemble refinement   

One major obstacle to the use of ER is the tendency of the MD simulation to excessively sample (*i.e.* locally ‘unfold’) the most disordered parts of the structure. To overcome this particular obstacle, DEN restraints (Schröder *et al.*, 2010 ▸, 2014 ▸) are now implemented within ensemble refinement, which act to restrain the simulation and enforce more local sampling. DEN restraints are an additional set of distance restraints placed on the atomic model to maintain its current geometry, which are then updated periodically during refinement; the effect of these restraints is that the atomic model is allowed to evolve over time, but as it is continually biased towards its current conformation this evolution is slow, and abrupt jumps through conformational space are discouraged. As implemented in ER, random restraint pairs are updated every 500 macrocycles during the simulation to prevent the initial choice of atoms involved in DEN restraints from biasing the sampling.

We demonstrate the effects of DEN restraints on three example structures (PDB entries 1uoy, 1ytt and 3k0n; Berman *et al.*, 2000 ▸, 2003 ▸; Olsen *et al.*, 2004 ▸; Fraser *et al.*, 2009 ▸; Burling *et al.*, 1996 ▸); the results of simulations with and without DEN restraints are shown in Table 1 ▸ (PDB entry 1uoy), Table 2 ▸ (PDB entry 1ytt) and Table 3 ▸ (PDB entry 3k0n), and the inclusion of DEN restraints leads to a marginal improvement in *R*_free_ in all cases. For details of how ER parameter optimizations were performed, see the supporting information. The implementation of DEN restraints creates more consistency in *R*_free_ values for different combinations of ER parameters (Supplementary Fig. S1), while still allowing the molecular-dynamics simulation to explore different conformations. The DEN restraints do not significantly affect the heterogeneity of the output structures, except in the most disordered side chains (Supplementary Fig. S2).

**Figure 1:**
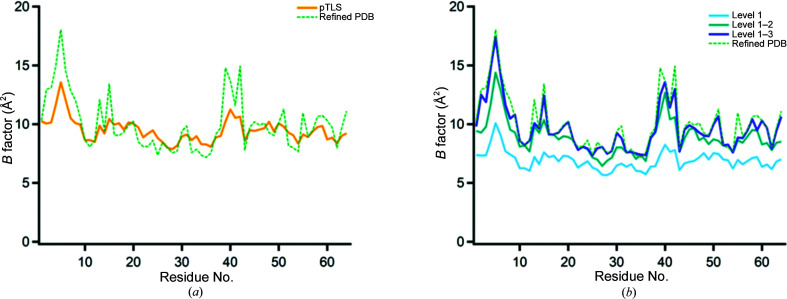
pTLS and ECHT *B*-factor profiles for PDB entry 1uoy. (*a*) Refined *B* factors of the input model and fitted pTLS *B*-factor profile. The fitted pTLS profile exhibits larger *B* factors than the input refined *B* factors over large parts of the model. (*b*) Profiles of combinations of disorder components from the ECHT disorder model. Disorder levels are (1) chain level, (2) secondary structure level and (3) residue level. Combinations of different components are always less than the total refined *B* factor for each atom, but approach these values as additional levels are added.

Secondly, the original underlying ER disorder model used TLS matrices optimized against the *B* factors of the C^α^ atoms of the input (classically refined) structure. These optimized TLS matrices are used to calculate a disorder contribution for atoms throughout the simulation. To avoid the generation of *B* factors that are overly biased towards the largest *B* factors of the structure, a fraction of the atoms with the smallest *B* factors are used (fraction pTLS). However, the pTLS approach will tend to produce an overestimation of the collective disorder components, since the pTLS *B* factors are generally too large for the least-disordered parts of the model (Fig. 1*a* ▸). This violates a central physical assumption of any disorder model: any partial disorder component should generally produce *B* factors for each atom that are less than (or equal to) the total *B* factor for that atom. This is required because all physical disorder components are constrained to be positive in size (or zero). The overestimation of disorder by the pTLS approach effectively drives the atoms with the lowest *B* factors to have zero-amplitude motions in the MD simulation, since any nonzero movement of the atom adds an additional disorder component to a *B* factor that is already too large. Therefore, using more physical disorder models should allow higher quality – but also more interpretable – models. The utilization of ECHT disorder models overcomes the limitations of the pTLS approach, since ECHT models contain components for all length scales of disorder: the optimized output components at each length scale are thus appropriately sized, and arbitrary combinations of the different levels will always give *B* factors less than (or equal to) the input refined *B* factors.

**Figure 2:**
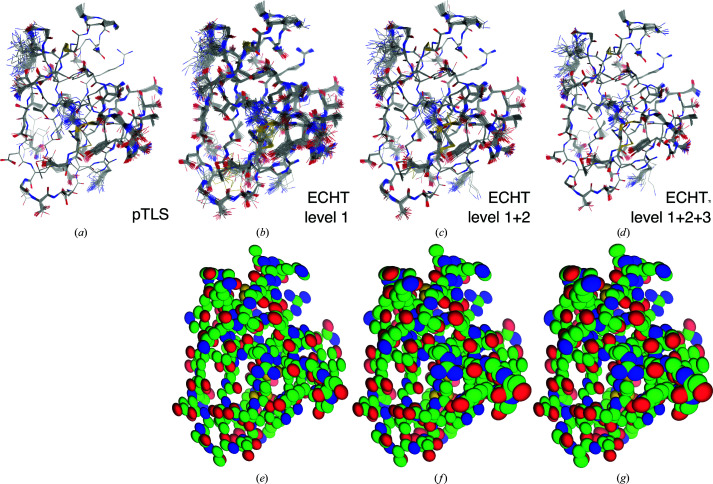
Ensemble refinements of PDB entry 1uoy with different *B*-factor models. (*a*) Ensemble refinement of PDB entry 1uoy using the pTLS model. Due to the systematic overestimation of the *B* factors in the pTLS model, the atomic fluctuations are forced to zero throughout the core of the structure. (*b*–*d*) Ensemble models obtained for (*e*–*g*) different components from the ECHT disorder model. (*b*, *e*) Chain-level disorder only (level 1), (*c*, *f*) sum of chain-level and secondary structure-level disorder components (level 1+2), (*d*, *g*) sum of chain-level, secondary structure-level and residue-level disorder components (level 1+2+3). As the amount of disorder increases in the ECHT disorder model from additional levels, the fluctuations in the atomic positions from the MD simulation decreases, and approach zero for atoms in the most ordered parts of the structure.

In this work, the ECHT disorder model is fitted to the classically refined structure, and different combinations of the chain-level, secondary structure-level and residue-level disorder components are used as input disorder models to ER (DEN restraints are used in all cases); we call this approach echtER. By using different combinations of the ECHT levels to parameterize the collective disorder in ER, the amount of disorder that the MD simulation must sample can be rationally selected (see, for example, Fig. 2 ▸). For example, by using the chain-level ECHT disorder component in ER, only the molecular disorder is modelled by a TLS contribution, with the result that the MD solution must sample all other intramolecular disorder; providing iteratively more levels from the ECHT model allows the sampling of the ER simulation to be systematically decreased. As more and more disorder components are added to the input disorder model, the fluctuations in the low-*B*-factor areas of the structure once more approach zero; however, in contrast to the pTLS approach, this zero-point motion is because the *B* factor is more appropriately estimated, rather than systematically overestimated.

The inclusion of more levels from the ECHT model leads to marginal, but systematic reductions in the *R*_free_ values for PDB entry 1uoy, and the combination of DEN + ECHT produces comparable *R* values to the DEN + pTLS approach (Table 1 ▸, Supplementary Fig. S3). These features are repeated for PDB entries 1ytt and 3k0n (Tables 2 ▸ and 3 ▸, Supplementary Figs. S4–S9), with the optimal model being achieved by including all three selected ECHT levels in the underlying disorder model; the exception to this is PDB entry 1ytt, where the *R*_free_ values are lowest when the ECHT level 1+2 model is used and the pTLS *R*_free_ values are significantly higher than the ECHT models, although this is possibly because this simulation had not fully converged (Supplementary Fig. S5). In the case of PDB entry 1ytt, the comparison of the pTLS profile and the ECHT level 1 profile is stark: although they are purportedly intended to estimate the same component, the pTLS approach attempts to attribute the disorder of the exterior residues to a chain-level motion, whereas the ECHT model largely partitions this disorder into both secondary-structure and residue components (Supplementary Fig. S4). In the case of PDB entry 3k0n, the new disorder models reproduce the previously observed heterogeneity in the active site (Supplementary Fig. S10), showing that neither DEN restraints nor ECHT disorder models degrade the quality of the simulations.

With DEN restraints and ECHT disorder models, the different structures have common characteristics between the parameter landscapes (Supplementary Figs. S3, S5, S8 and S11): the quality of the simulations generally exhibits a strong dependence on the relaxation time (TX) and a weak orthogonal dependance on the X-ray weight (WX). This allows a linear search pattern where TX is varied for a fixed WX, and then WX is varied for the optimal TX. Utilizing a parameter search formed of two linear searches is much less computationally intensive than the full 2D grid search. The utilization of ECHT disorder models also eliminates the need to optimize one ER input parameter: the pTLS fraction. This was always a somewhat arbitrary nuisance variable and needed to be determined empirically as the value that retrospectively led to the lowest *R*_free._ This significantly increased the grid search space by adding another continuous variable dimension in addition to the WX and TX variables, greatly increasing the total computational load. A weak correlation of pTLS with *R*_free_ was observed previously, but it caused systematic changes to sampling, with a larger pTLS reducing global sampling and vice versa (Burnley *et al.*, 2012 ▸). The utilization of ECHT disorder models allows a rational choice of input disorder model based on the sampling that the user chooses to perform; any element of disorder that the user does not wish to sample can be included in the input disorder model.

## 3. Application to SARS-CoV-2 main protease   

To further explore the differences in dynamics that can be observed through ER, we applied the method to a high-resolution structure of the SARS-CoV-2 main protease (PDB entry 7k3t) re-refined with *PDB-REDO* (Joosten *et al.*, 2009 ▸, 2014 ▸). Once more, the use of echtER produced lower *R*_free_ values than pTLS refinements, and once more the difference is marginal (Table 4 ▸). Both approaches of ER produced lower *R* factors than the single-model refinements. The main protease displays multiple flexible loops, and the structural heterogeneity in these loops changes significantly depending on the underlying disorder model. Notably, the larger structure also requires much longer relaxation times than for the smaller structures (Supplementary Fig. S11); this potentially reflects fundamentally larger disorder in the data that requires longer timescales to explore. The behaviour of this structure under different parameter combinations also implies that even longer relaxation times could lead to even lower *R*_free_ values.

**Figure 3:**
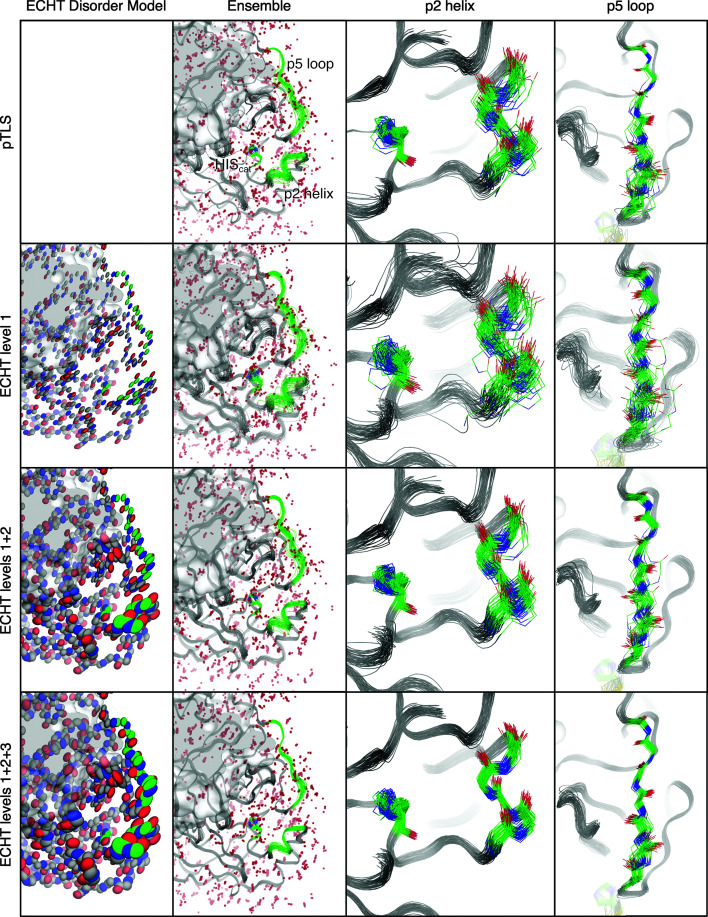
Backbone variation in ensemble refinements of PDB entry 7k3t with different underlying disorder models. An equivalent figure with side chains is shown in Supplementary Fig. S12. Structures shown are as in Table 4 ▸. Rows are labelled with the disorder model used. Column 1: the input disorder for the ECHT disorder models. Column 2: the output ensemble in the area surrounding the active site (the catalytic His residue is indicated), including the p2 helix and the p5 loop. Column 3: the disorder in the p2 helix systematically decreases as more disorder is added to the underlying disorder model, but the ensembles are all qualitatively similar, confirming that the helix, although flexible, adopts only one distinct conformation. Column 4: the pTLS model suppresses disorder at the top of the p5 loop. Different ECHT disorder models systematically increase disorder in the *B* factors and correspondingly decrease disorder in the obtained ensemble. The ECHT level 1 ensemble is the most physically interpretable ensemble, since this contains all of the disorder in the ensemble apart from collective molecular motions. In the ECHT level 1+2 ensemble and the ECHT level 1+2+3 ensemble some of this disorder is now hidden in the *B* factors, and only the complex residue motions remain.

Structural differences are most clearly shown by two secondary-structure elements where disorder has previously been observed: the p2 helix and the p5 loop (Kneller *et al.*, 2020 ▸; Ebrahim *et al.*, 2021 ▸). The p2 helix exhibits a collective disorder pattern in ECHT analyses, implying that the helix may undergo rigid-body-like motions, while the p5 loop shows internal flexibility (Pearce & Gros, 2021 ▸). These observations are mirrored by the different ensembles that are obtained (Fig. 3 ▸, Supplementary Fig. S12), where the p2 helix primarily maintains the same configuration whilst undergoing collective displacements, whilst for the p5 loop multiple conformations are sampled.

The differences between the pTLS refinements and the echtER refinements highlight how structural information is lost when using nonphysical disorder components for the underlying disorder model. By applying what is effectively a strong but inaccurate Bayesian prior characterizing the disorder of the molecule, the pTLS model suppresses disorder in key regions of the molecule, which precludes structural variations in neighbouring residues. This is exemplified by disorder in the p5 loop: in the pTLS optimization the top of the loop shows very limited heterogeneity, whilst the bottom of the loop shows significant heterogeneity. However, in the echtER refinements this loop shows variation along the entirety of its length, although the conformational heterogeneity is once more concentrated in the bottom of the loop. This changes the interpretation of the output model, and with echtER yields a very different structural analysis in which all of the loop is shown to be flexible. Additionally, although the top of the loop is less disordered with the pTLS model, variation in the positions of residues may be required for subsequent conformational changes in neighbouring residues; restricting the positional variation of some residues may therefore cause knock-on effects for connected residues and preclude conformational changes and therefore conformational sampling of different states, or lead to strains on the geometry of the model.

The behaviour in disordered parts of the protein can be seen in the C-terminus of the protein, which exhibits significant disorder, as already indicated by the inflated atomic *B* factors in the input model (Supplementary Fig. S13). Even with DEN restraints this region is highly mobile, suggesting that DEN restraints do allow disordered regions to be disordered, rather than inappropriately suppressing disorder/motion. Once more, the increased disorder in the *B* factors for the ECHT 1+2+3 model leads to the least disorder in the output ensemble; beyond this difference, the output ensembles for the different echtER models are qualitatively similar.

## 4. Discussion   

The incorporation of DEN restraints into ER stabilizes simulations and enables more efficient exploration of the search space of molecular conformations, while still permitting disorder in disordered regions. The additional usage of ECHT disorder models in ER – echtER – generates more realistic and physical simulations, improving on the ensembles from the pTLS approach that lead to an unpredictable (mis)characterization of the heterogeneity in different structural components. The usage of ECHT disorder models allows the systematic probing of structural disorder by allowing well defined disorder components to be purposefully selected and allowing the modeller to choose the level of complexity that they wish to enforce on the model. The well defined nature of ECHT disorder models may prove to be crucial if we wish to directly compare conformational ensembles from different data sets of the same system, such as a recent study that used ER to study the conformational ensembles of the SARS-CoV-2 protease as a function of temperature (Ebrahim *et al.*, 2021 ▸).

For the investigation of correlated motions and dynamics, it seems clear that only the large-scale disorder components should be used (*i.e.* ECHT level 1, representing collective molecular disorder), as this allows interplay between the different scales of disorder; however, it is equally clear that the optimum *R*_free_ is most often achieved when we supply levels up to residue disorder (*i.e.* ECHT level 1+2+3), and therefore encourage conformational sampling of only local anharmonic motions that cannot be well represented by typically used descriptions of *B* factors. This automatic sampling of anharmonic oscillations is a major advantage of ensemble refinement over single-state atomic models, similarly to other methods that are built explicitly on the fundamental and inescapable anharmonicity of macromolecular motions (Ginn, 2021 ▸). As more disorder is partitioned into the ECHT model (*i.e.* as more levels are added) less remains to be sampled during the MD simulation and the resultant ensemble is visibly dampened. This feature may be useful for low-resolution data sets where reducing the conformational sampling is necessary to reduce overfitting. However, determining the residue component of disorder may also become unreliable where a residue is poorly defined in the input classically refined model (for example for disordered loops), and therefore in general we recommend users start with an ECHT model representing chain and secondary-structure disorder (ECHT level 1+2) but explore the other levels where appropriate.

The echtER workflow is therefore as follows: the ECHT model is fitted against the classically refined input model, the desired combinations of ECHT levels are chosen and finally ER simulations are run for each combination of ECHT levels to optimize the TX and WX parameters. Previously, ER required a computationally intensive grid search over three continuous parameters: pTLS, WX and TX. The use of ECHT models replaces the continuous pTLS parameter with the combinations of discrete ECHT levels, thereby simplifying the complexity of the parameter space (the time required to parameterize an ECHT model is negligible compared wih the runtime of an ER simulation). Additionally, we have found that a computationally efficient route to performing the WX–TX grid search is to optimize the TX parameter for the default WX, and then optimize WX for the identified TX; if necessary, this approach can be used to further reduce the total number of computationally intensive ER runs that need to be performed.

With the improved sampling presented here, the future improvements of the ER approach must now come from the use of more sophisticated force fields, such as Amber (Moriarty *et al.*, 2020 ▸), as rightly noted in other work (Ebrahim *et al.*, 2021 ▸). The improvements listed in this work, along with the proposed future directions, should overcome much of the valid criticism of ER models, such as poor geometry, and make ER a standard option for crystallographic structure determination. One of the great advantages of ER is the removal of bias on the part of the modeller, instead replacing our best single model with a Bayesian ensemble of structures. The output models, particularly those achieved using a minimal disorder model (*i.e.* ECHT level 1), serve to remind us that macromolecules are flexible dynamic molecules, and force us to reconcile our typical views of macromolecules as a single static conformation with reality, which is that a large amount of variation is encoded, and subsequently hidden, within atomic *B* factors.

## 5. Availability   

The ER approach is available within the *Phenix* package (Liebschner *et al.*, 2019 ▸); providing custom disorder models (*i.e.* using echtER) requires update 4302 or later. The ECHT model, and a script giant.swerp (**sw**eep **e**nsemble **r**efinement **p**arameters) for automating the generation of parameter sweeps, are provided in the *panddas* package (https://pandda.bitbucket.io). All ER simulations, including ECHT outputs, have been uploaded to Zenodo (https://doi.org/10.5281/zenodo.5226789). Along with this work, Burnley & Gros (2013 ▸) contains some guidance on the preparation of models for use with the ER method.

## Supplementary Material

Supporting Information, Supplementary Table and Figures. DOI: 10.1107/S2059798321010044/qn5002sup1.pdf

## Figures and Tables

**Table 1:** Refinement results for PDB entry 1uoy (resolution 1.5 Å) Structures were re-refined with *phenix.refine* prior to ER to yield comparable *R* factors. The ECHT model was fitted to the re-refined model. The re-refined structure was used as input for all ER runs. The model with the lowest *R*_free_ for each parameter sweep is shown.

Refinement	DEN restraints	Disorder model	*R*_work_/*R*_free_	WX	TX	pTLS
Deposited	—	—	0.164/0.185	—	—	—
*phenix.refine*	—	—	0.130/0.169	—	—	—
ER	No	pTLS	0.113/0.148	0.25	0.5	0.9
ER	Yes	pTLS	0.111/0.147	2	1	0.9
ER	Yes	ECHT level 1	0.113/0.153	0.5	1	—
ER	Yes	ECHT level 1+2	0.109/0.147	0.25	2	—
ER	Yes	ECHT level 1+2+3	0.110/0.147	0.5	2	—

**Table 2:** Refinement results for PDB entry 1ytt (resolution 1.8 Å) Details are as in Table 1 ▸.

Refinement	DEN restraints	Disorder model	*R*_work_/*R*_free_	WX	TX	pTLS
Deposited	—	—	0.185/0.206	—	—	—
*phenix.refine*	—	—	0.161/0.216	—	—	—
ER	No	pTLS	0.178/0.221	1	0.125	0.5
ER	Yes	pTLS	0.177/0.220	0.25	0.125	0.7
ER	Yes	ECHT level 1	0.143/0.191	4	1	—
ER	Yes	ECHT level 1+2	0.150/0.190	8	0.5	—
ER	Yes	ECHT level 1+2+3	0.152/0.196	0.25	0.5	—

**Table 3:** Refinement results for PDB entry 3k0n (resolution 1.4 Å) Details are as in Table 1 ▸.

Refinement	DEN restraints	Disorder model	*R*_work_/*R*_free_	WX	TX	pTLS
Deposited	—	—	0.122/0.160	—	—	—
*phenix.refine*	—	—	0.124/0.150	—	—	—
ER	No	pTLS	0.105/0.128	1	2	0.5
ER	Yes	pTLS	0.100/0.125	0.50	4	0.3
ER	Yes	ECHT level 1	0.099/0.134	1	8	—
ER	Yes	ECHT level 1+2	0.101/0.124	0.25	4	—
ER	Yes	ECHT level 1+2+3	0.101/0.123	1	8	—

**Table 4:** Refinement results for PDB entry 7k3t (resolution 1.2 Å) Details are as in Table 1 ▸, except that the ECHT model was fitted to the *PDB-REDO* refined structure.

Refinement	DEN restraints	Disorder model	*R*_work_/*R*_free_	WX	TX	pTLS
Deposited	—	—	0.171/0.187	—	—	—
*phenix.refine*	—	—	0.146/0.173	—	—	—
ER	Yes	pTLS	0.139/0.169	8	8	0.3
ER	Yes	ECHT level 1	0.136/0.176	8	8	—
ER	Yes	ECHT level 1+2	0.139/0.171	8	8	—
ER	Yes	ECHT level 1+2+3	0.143/0.167	8	8	—
