# Supplementary material for: Improving sampling of crystallographic disorder in ensemble refinement
Source: Acta Crystallogr D Struct Biol. 2021 Oct 20;77(Pt 11):1357–64. doi: 10.1107/S2059798321010044 (PMC8561732; doi:10.1107/S2059798321010044)
Supplement: Supplementary file 1 [file d-77-01357-sup1.pdf]

STRUCTURAL BIOLOGY

Volume 77 (2021)

Supporting information for article:

Improving sampling of crystallographic disorder in ensemble refinement

Nicoleta Ploscariu, Tom Burnley, Piet Gros and Nicholas M. Pearce

S1. Ensemble Refinement model generation

S1.1. ECHT Model Parameterisation

ECHT model characterisations were performed on the re-refined structures using the default parameters. ECHT outputs are included as part of the supplementary data (see availability).

S1.2. Ensemble Refinement Model Preparation

S1.2.1. 1UOY

The model for ensemble refinement was prepared by refining the model from PDB_REDO with phenix.refine for 8 cycles using standard refinement options, with all atoms except water using anisotropic B-factors.

S1.2.2. 1YTT

The model for ensemble refinement was prepared by refining the model from PDB_REDO with phenix.refine for 8 cycles using the standard refinement options, with all atoms except water using anisotropic B-factors. Stable refinement of the Yb atoms required restraints to prevent disassociation of the Yb atoms from the protein. Initial simulations were performed with the initial model in which Yb atoms had full occupancy (1.0) and different restraints were tested. The best results were obtained using weight = 0.007 and slack = 0.6. The initial model was also re-refined where the occupancy of Yb atoms was additionally refined and the values found were updated in the input file for ER. Refined occupancies for the Yb atoms were 1.00 (A1), 0.81 (A2), 0.9 (B1) and 0.8 (B2).

S1.2.3. 3K0N

The model for ensemble refinement was prepared by refining the model from PDB_REDO with phenix.refine for 8 cycles using the standard refinement options, with all atoms except water using anisotropic B-factors.

For ECHT parameterisation, secondary structure using the DSSP algorithm failed to run on this structure, so secondary structure definitions were taken from the annotated PDB structure, and supplied manually to ECHT.

After initial ensemble refinement simulations, high positive peaks were identified in the difference map in the neighbourhoods of some S atoms for Met and Cys residues. These arose due to the insertion by the ER algorithm of water atoms into the space temporarily-vacated by the protein residues. In these cases, these residues are embedded in the core of the structure and the waters therefore cannot be ejected, leading to the identified difference density. To prevent the erroneous

water placement, the S atoms in the identified cysteine and methionine residues were restrained to their initial positions using weight = 0.005 slack = 1.0.

S1.2.4. 7K3T

The model for ensemble refinement was prepared by refining the model from PDB_REDO with phenix.refine using group occupancies for the bound DMSO molecules. This model was then resubmitted to the PDB_REDO server and the resulting model was used as input to ER.

S1.3. Ensemble Refinement Parameterisation

When DEN restraints are used, they are applied with a weight of 30 as per the recommended PHENIX defaults. In the case where an ECHT disorder model is not supplied as input to the program, a pTLS value is provided instead. Parameter sweeps for Ensemble Refinement parameters as shown in Table S1. For each ECHT disorder model, a grid search was performed over the set of TX and WX parameters; for pTLS models, a grid search was performed over the set of pTLS, TX and WX parameters. In both cases, statistics and parameters presented for each data set are for the parameter combination with the lowest R-free. Exact details of parameter files and commands uses can be found in the supplementary data (see availability).

Table S1 Ensemble Refinement Sweep Parameters. A grid search was performed using all combinations of the below parameters (including pTLS for pTLS runs, otherwise using the stated ECHT model as input). Parameters marked with an asterisk (*) were not run for 7k3t.

Ensemble Refinement Variable	Sweep Parameters
**WX**; X-ray weight	0.25, 0.5, 1, 2, 4, 8
**TX**; Relaxation time	0.0625*, 0.125*, 0.25, 0.5, 1, 2, 4, 8
**pTLS**; fraction-TLS	0.3, 0.5, 0.7, 0.9

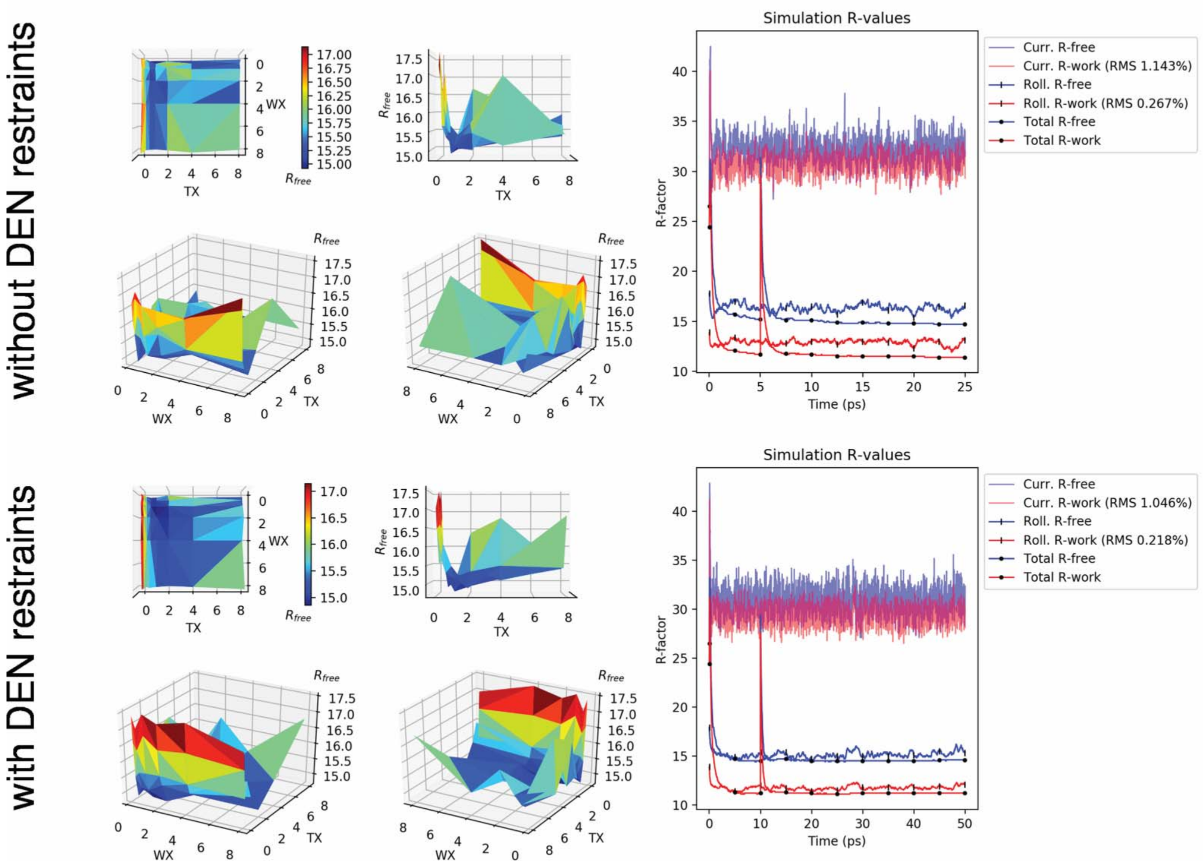

Figure S1 ER parameter sweep for 1UOY using pTLS with and without DEN restraints. Left-hand column shows the R-free for simulations with different parameter combinations of TX (relaxation time) and WX (X-ray weight). Right-hand column shows the R-values of the optimal simulation. There is a burn-in period at the beginning of each simulation. The instantaneous R-values (current model), rolling R-values (averaged over the relaxation time), and total R-values (averaged over the whole simulation) are shown. For pTLS runs, only the graph for the pTLS value corresponding to the R-free minimum is shown (pTLS=0.9).

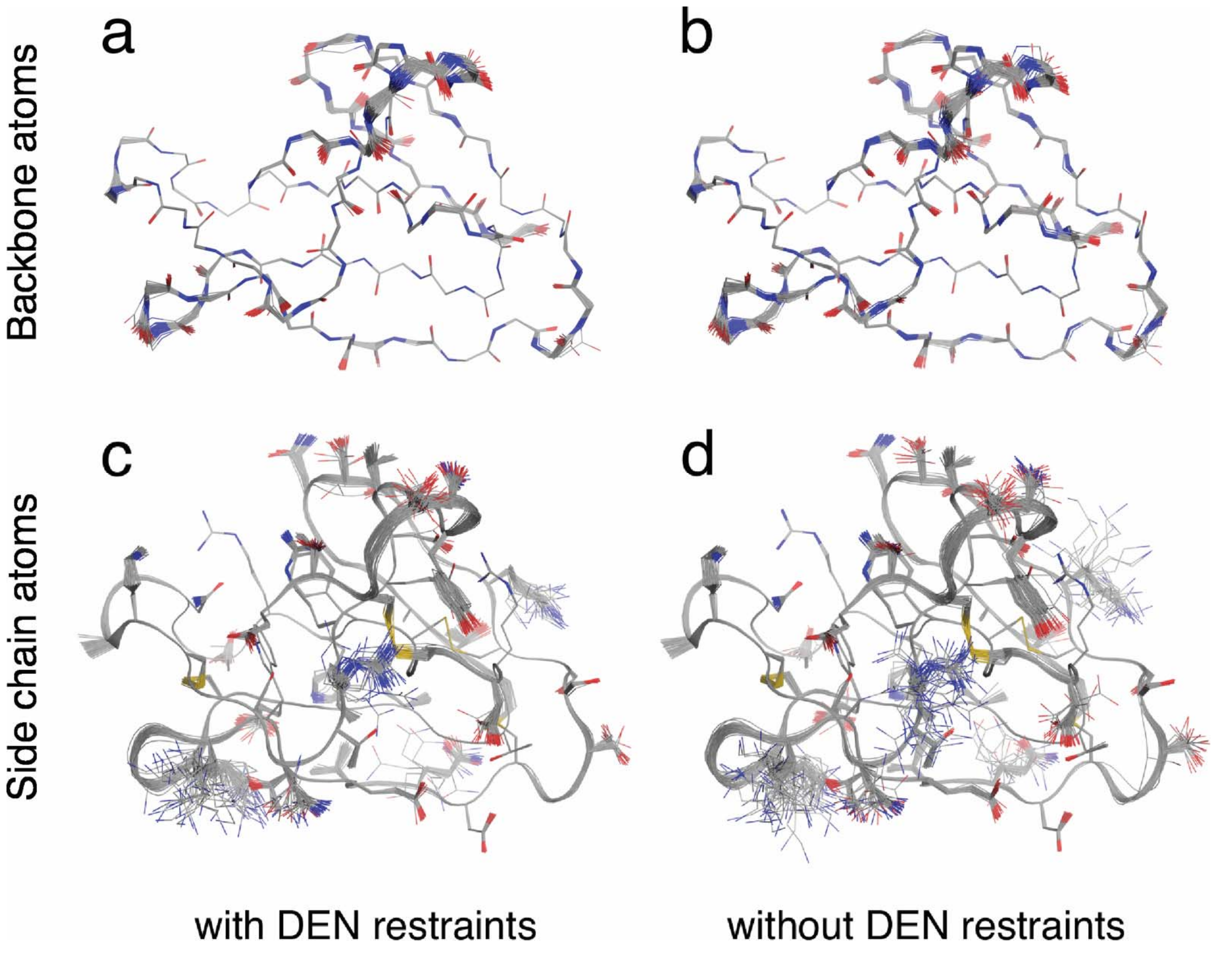

Figure S2 Ensemble refinements of 1UOY using pTLS with and without DEN restraints. Structures as those presented in Table 1. (a,c) Ensemble refinement with DEN restraints. (b,d) Ensemble refinement without DEN restraints. (a,b) Backbone atoms only, stick representation. (c,d) Backbone shown in cartoon tube representation with sidechains shown as sticks. The ensembles are highly similar, with the only notable differences in the lysine sidechains; thus, the DEN restraints only affect highly disordered regions of the structure and prevent excessive sampling.

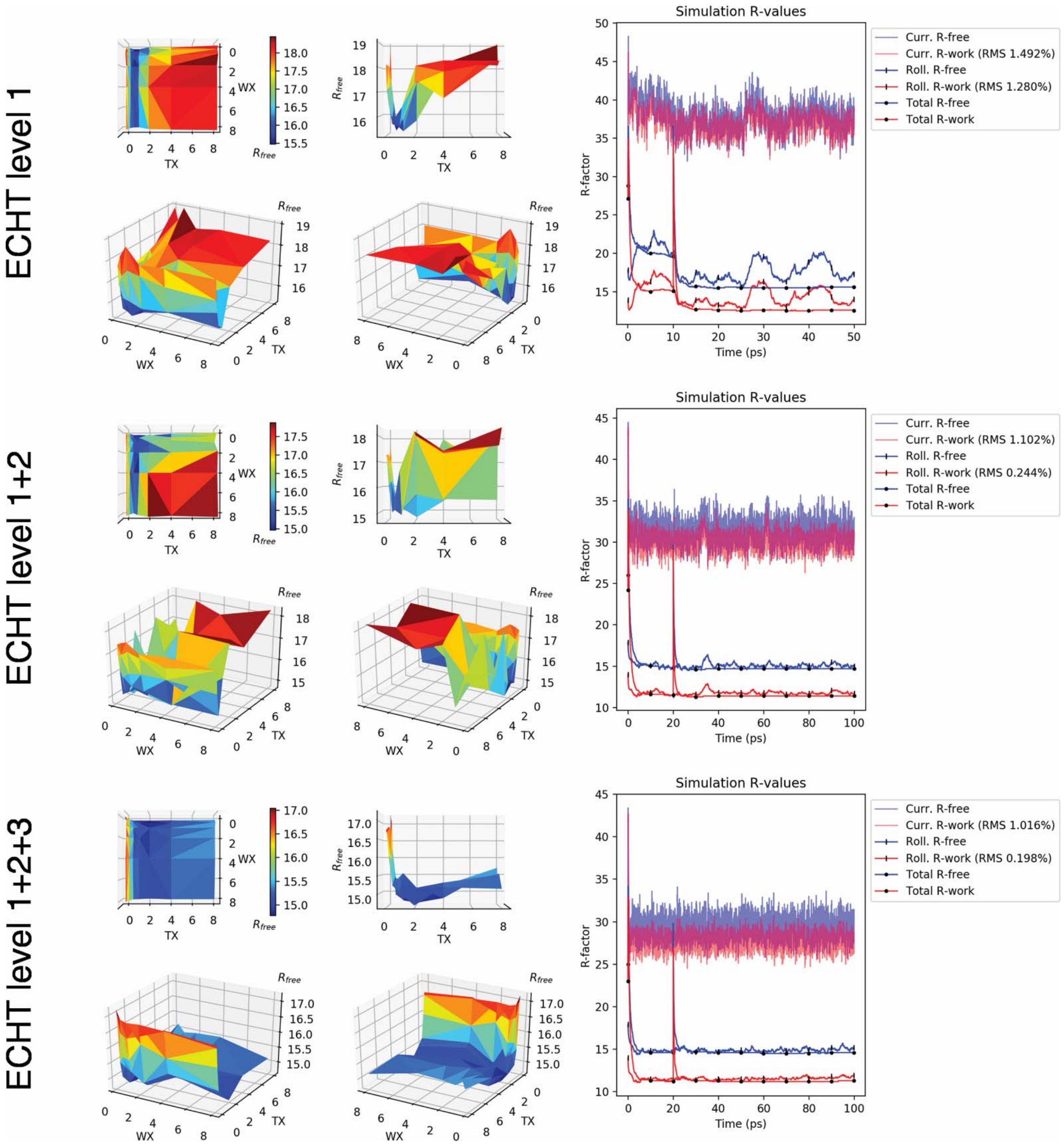

Figure S3 ER Parameter sweep for 1UOY with different disorder models from ECHT analysis**.** Layout as per Figure S1. ECHT levels are (1) chain level, (2) secondary structure, and (3) residue level. The optimisation landscapes form a characteristic trough for each disorder model.

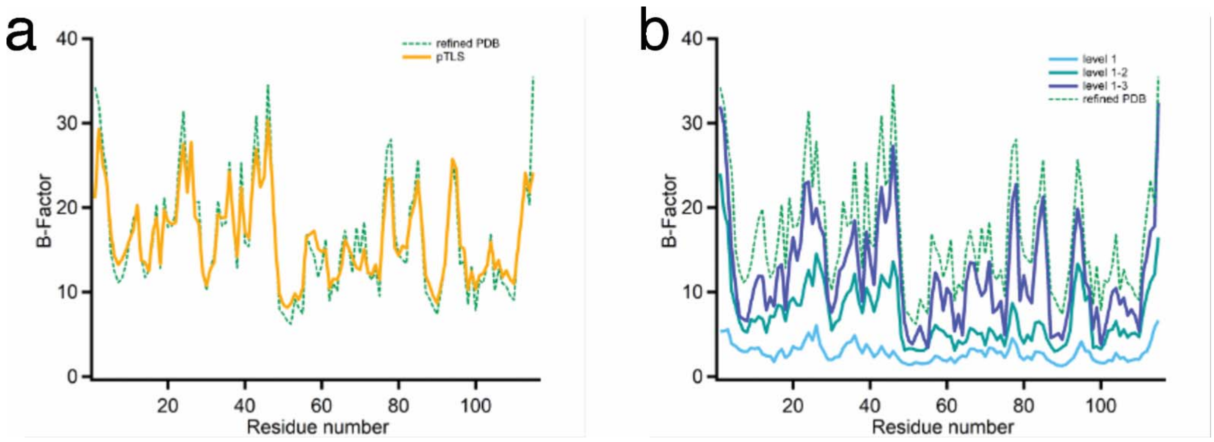

Figure S4 pTLS and ECHT B-factor profiles for 1YTT. Layout and details as per Figure 1. ECHT levels are (1) chain level, (2) secondary structure, and (3) residue level. Although the pTLS component seems to match the total B-factor trace, this is only the average per residue. The difference between the pTLS component and the chain-component of the ECHT model (level 1), which are naively meant to represent the same motion, is striking.

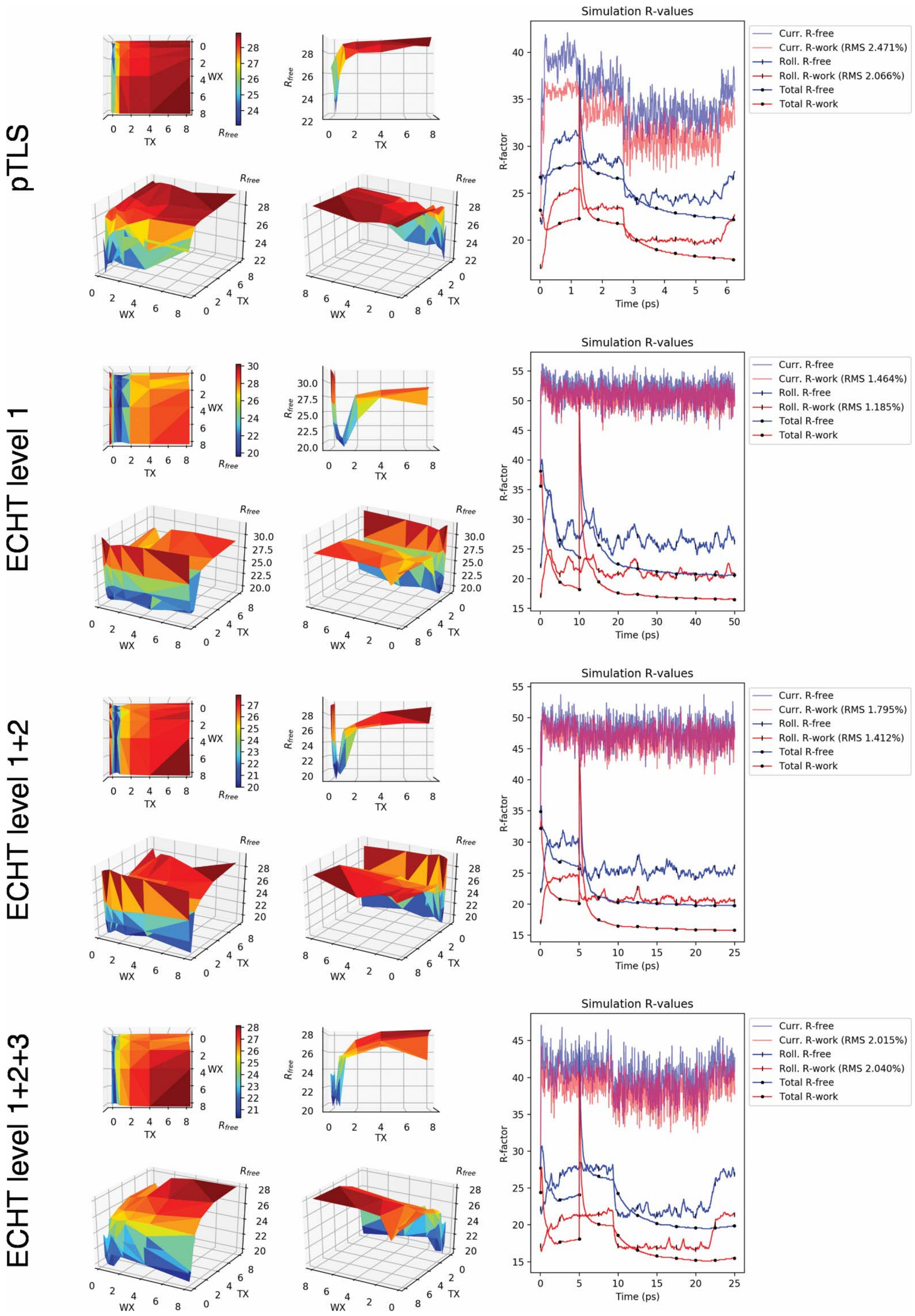

Figure S5 ER parameter sweep for 1YTT using pTLS and ECHT disorder models. Layout as per Figure S1. ECHT levels are (1) chain level, (2) secondary structure, and (3) residue level.

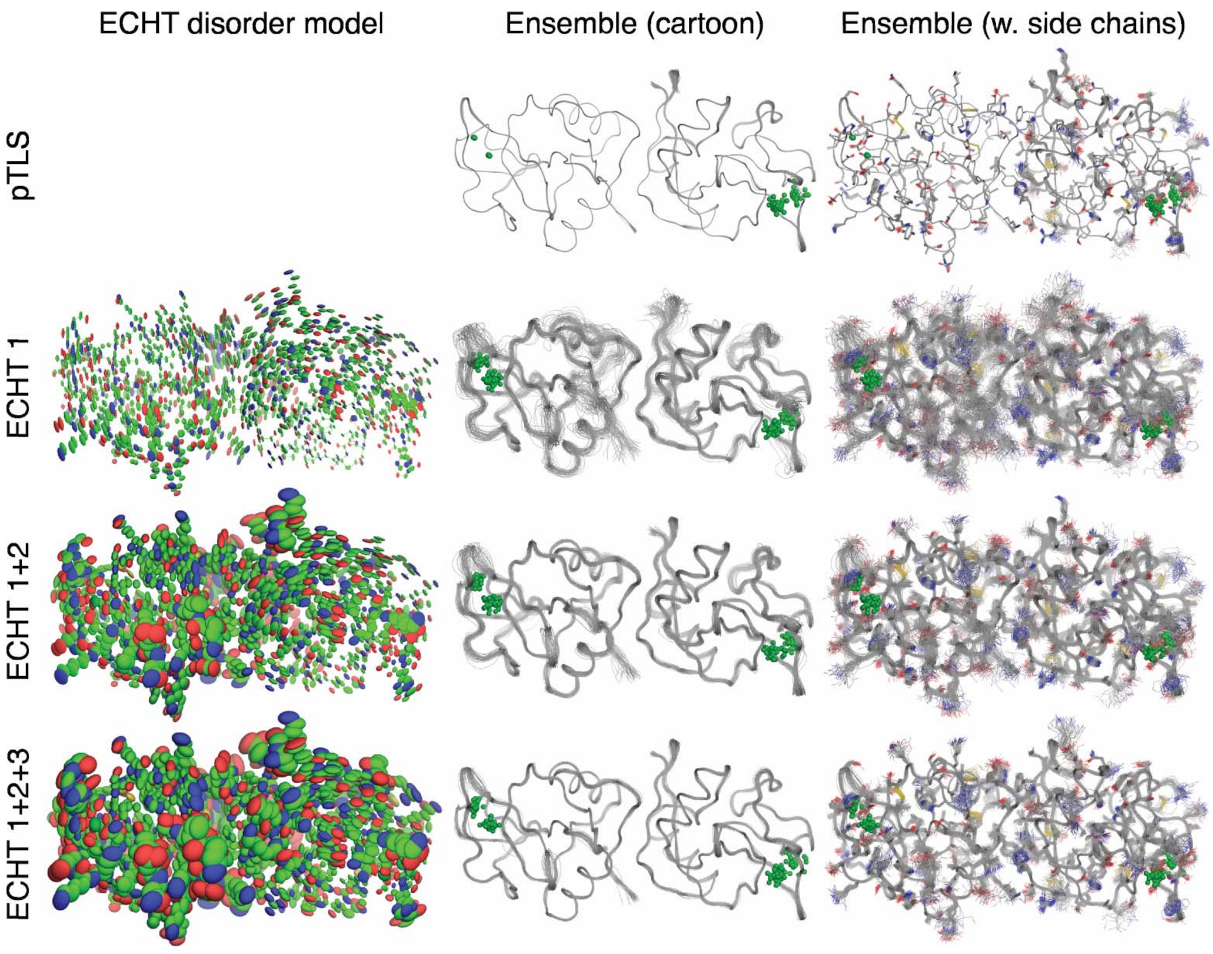

Figure S6 Ensemble Refinements of 1YTT with different B-factor models. Rows are labelled with the disorder model used: ECHT levels are (1) chain level, (2) secondary structure, and (3) residue level. (1st column) ECHT model disorder components used as input to ER. (2nd column) Output ensemble in cartoon tube representation, Yb atoms as spheres. (3rd column) As 2nd column but with sidechain atoms shown as sticks.

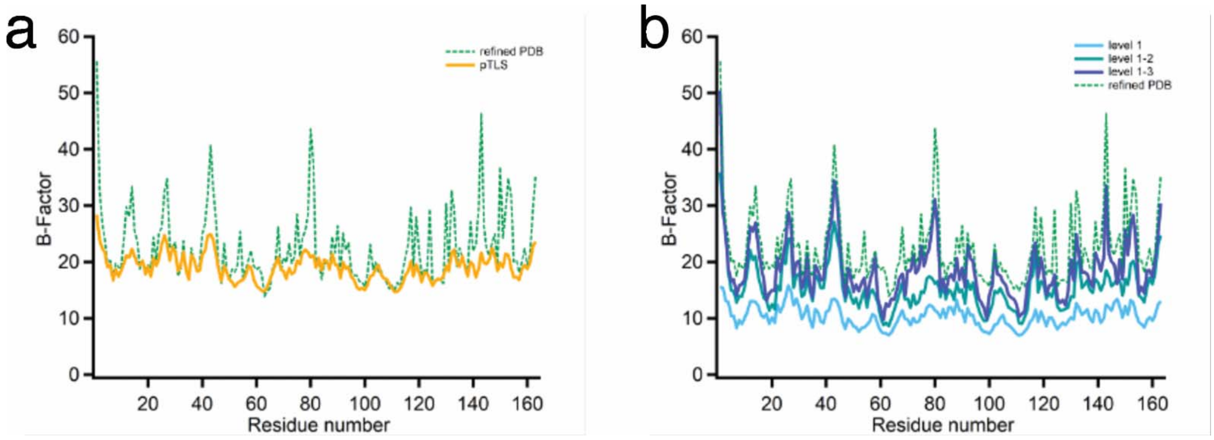

Figure S7 pTLS and ECHT B-factor profiles for 3K0N. Layout and details as per Figure 1. ECHT levels are (1) chain level, (2) secondary structure, and (3) residue level.

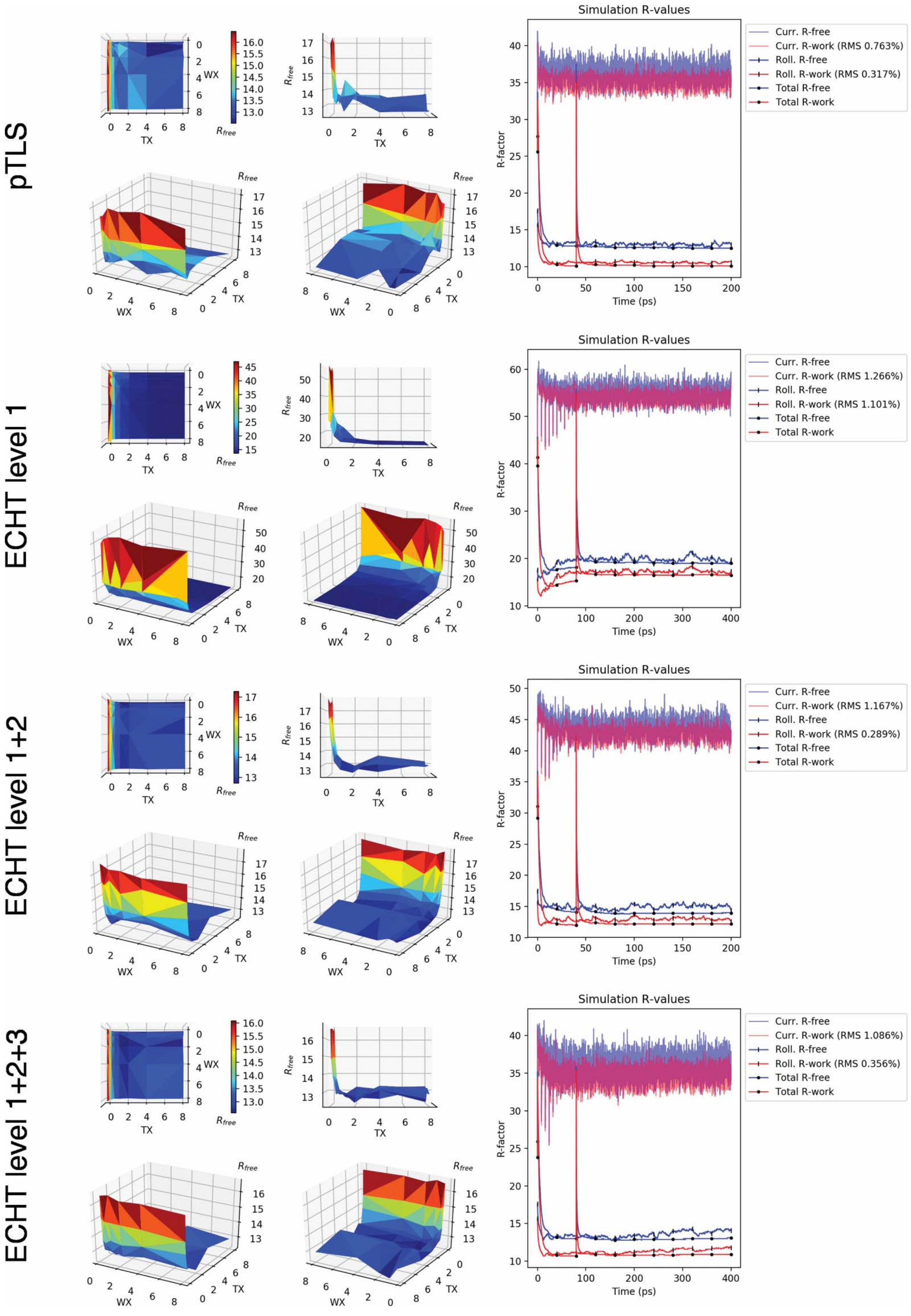

Figure S8 ER parameter sweep for 3K0N using pTLS and ECHT disorder models. Layout as per Figure S1. ECHT levels are (1) chain level, (2) secondary structure, and (3) residue level.

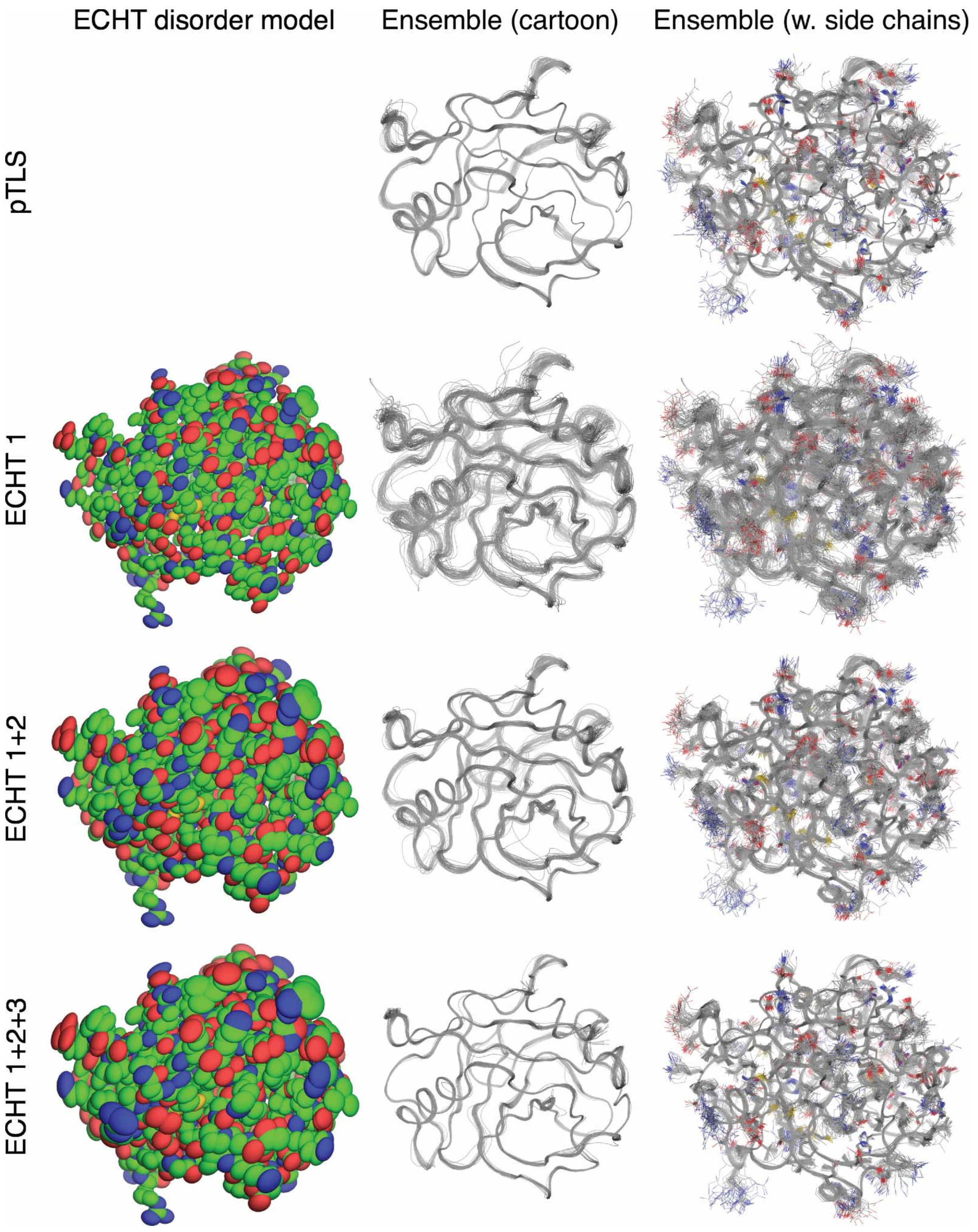

Figure S9 Ensemble refinements of 3K0N with different B-factor models. Rows are labelled with the disorder model used: ECHT levels are (1) chain level, (2) secondary structure, and (3) residue level. Layout as per Figure S6.

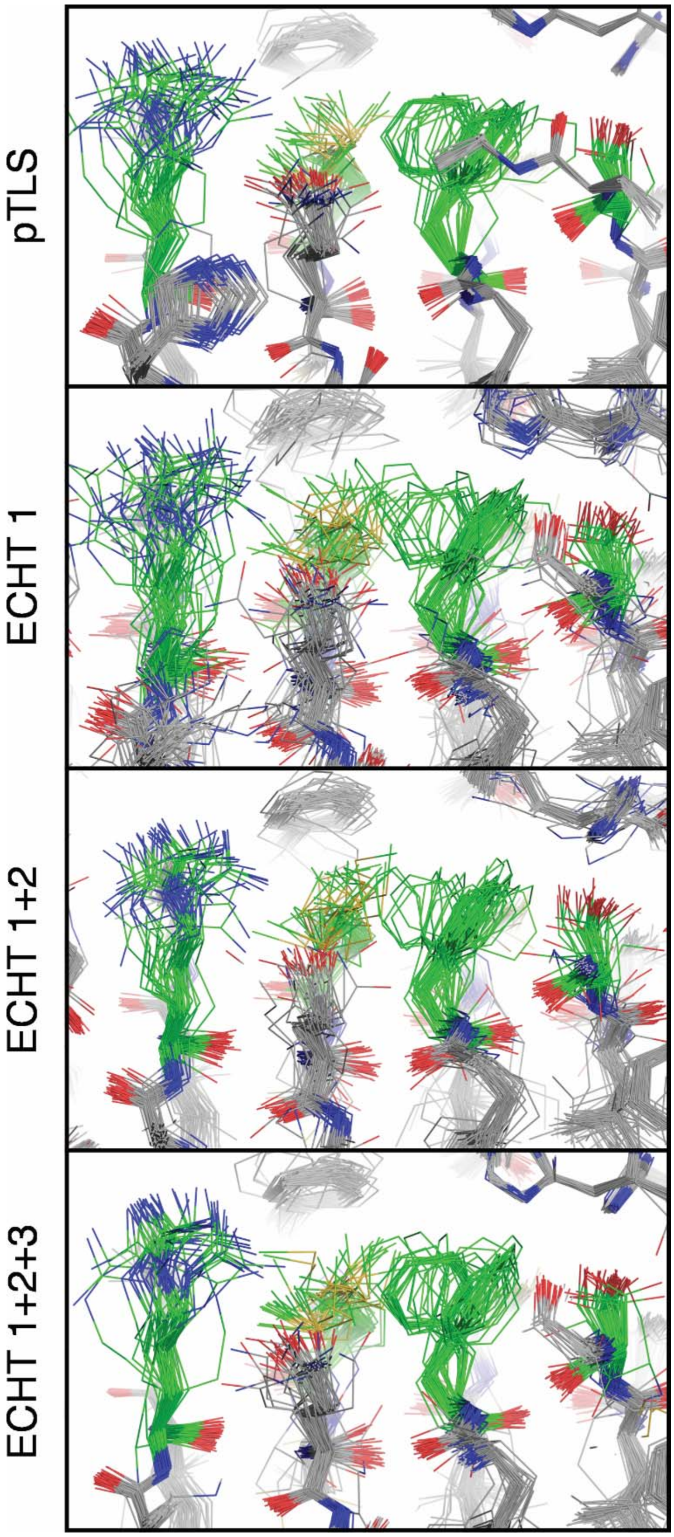

Figure S10 Active site network in 3K0N ensemble refinements. Rows are labelled with the disorder model used: ECHT levels are (1) chain level, (2) secondary structure, and (3) residue level. The heterogeneity of residues ARG55, MET61, SER99 and PHE113 is broadly conserved between the different disorder models, although the occupancy ratio of the two states varies.

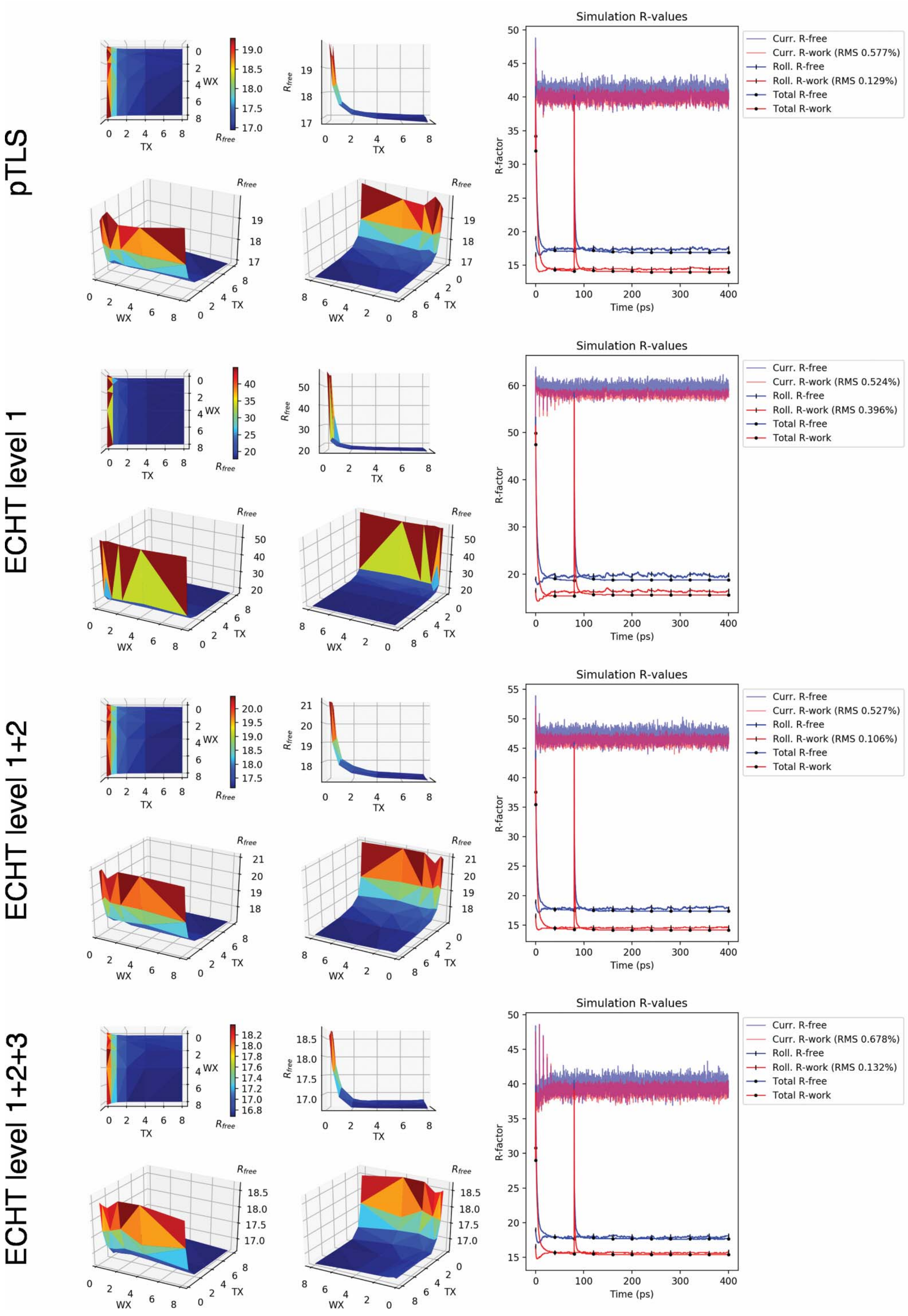

Figure S11 ER parameter sweep for 7K3T using pTLS and ECHT disorder models. Layout as per Figure S1. ECHT levels are (1) chain level, (2) secondary structure, and (3) residue level.

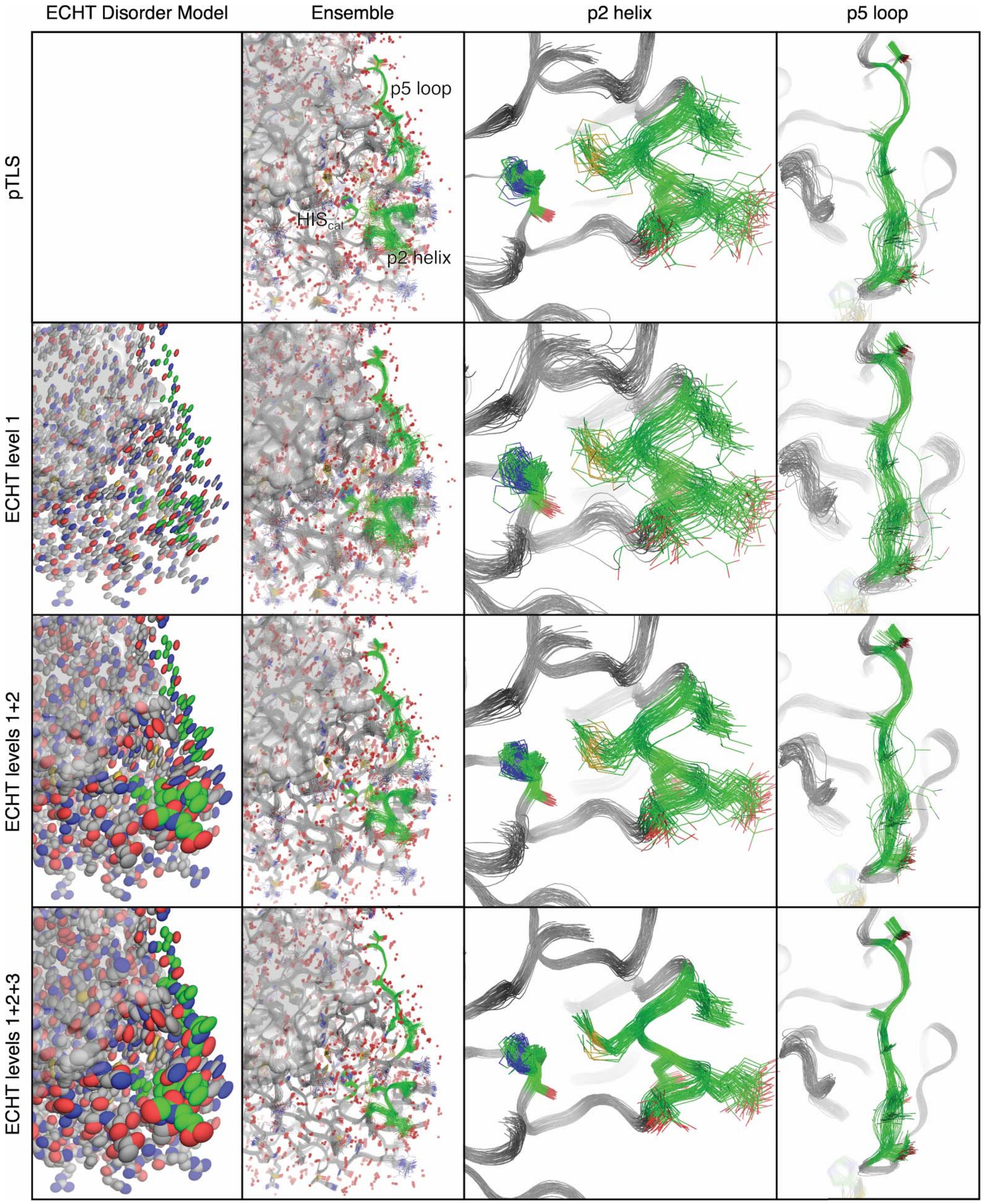

Figure S12 Sidechain variation in 7K3T for different underlying disorder models. Details as per Figure 3, but with sidechains shown as sticks and backbone shown as cartoon loop. ECHT levels are (1) chain level, (2) secondary structure, and (3) residue level.

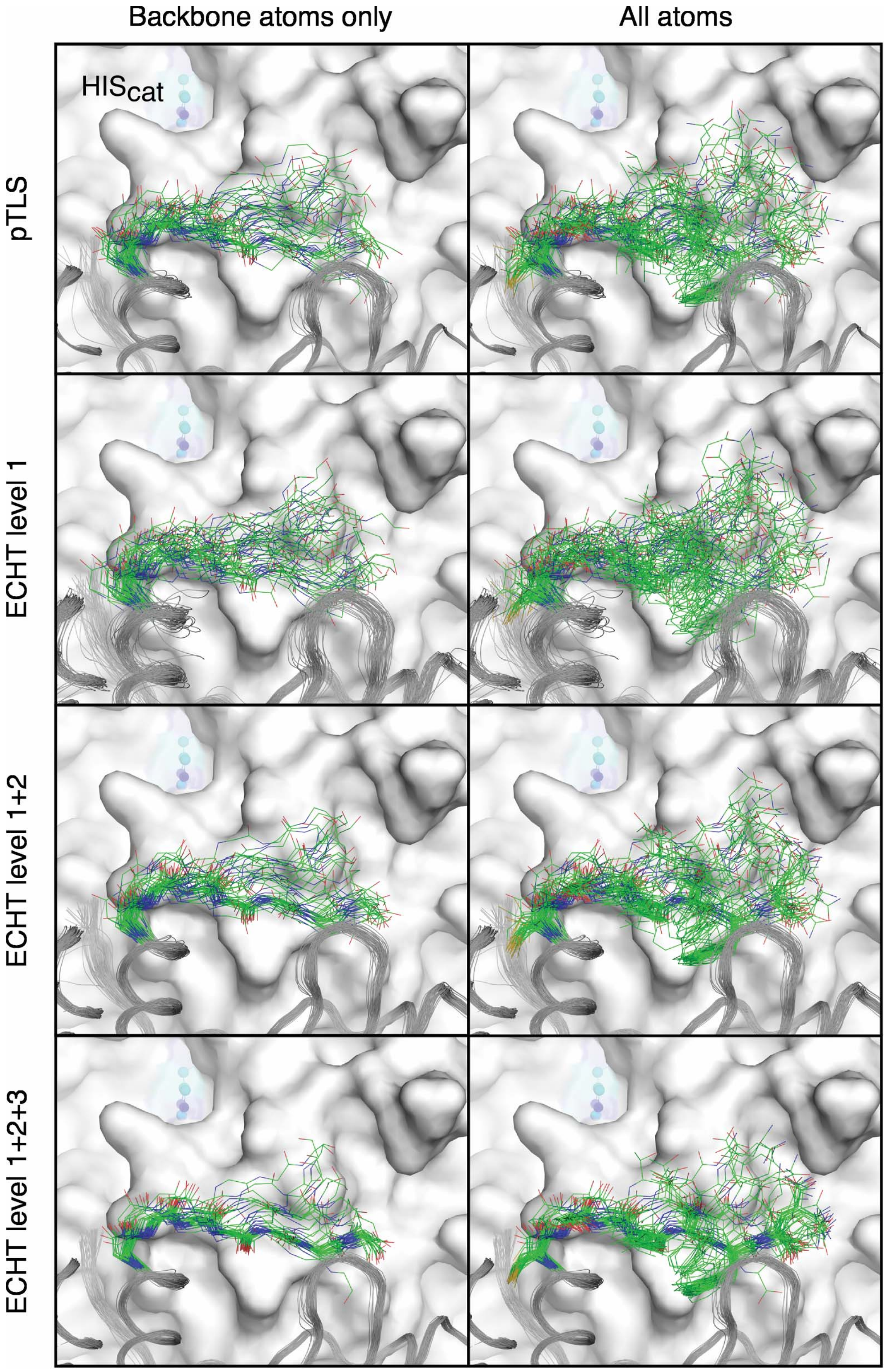

Figure S13 C-terminal disorder in 7K3T for different underlying disorder models. Rows are labelled with the disorder model used: ECHT levels are (1) chain level, (2) secondary structure, and (3) residue level. (1st column) Backbone as sticks for the C-terminus. (2nd column) also showing sidechains. Disorder in the C-terminal region is significant, but still varies systematically with the addition of additional ECHT levels. The proximity of the catalytic site (indicated) means that c-terminus behavior may be important for binding.
